# The effect of *Apolipoprotein E4* on cognitive function in Parkinson’s disease: A structural MRI study in the PPMI cohort

**DOI:** 10.1371/journal.pone.0341240

**Published:** 2026-01-20

**Authors:** Angenelle Eve Rosal, Edgardo Torres-Carmona, Sarah L. Martin, Isabelle Boileau, Ariel Graff-Guerrero, Antonio P. Strafella

**Affiliations:** 1 Brain Health Imaging Centre, Centre for Addiction and Mental Health, Toronto, Ontario, Canada; 2 Institute of Medical Science, Temerty Faculty of Medicine, University of Toronto, Toronto, Ontario, Canada; 3 Multimodal Imaging Group, Brain Health Imaging Centre, Centre for Addiction and Mental Health, Toronto, Ontario, Canada; 4 Department of Psychiatry, Centre for Addiction and Mental Health, Toronto, Ontario, Canada; 5 Edmond J. Safra Parkinson Disease Program, Neurology Division, Toronto Western Hospital & Krembil Brain Institute, University Health Network, University of Toronto, Toronto, Ontario, Canada; University of Thessaly Faculty of Medicine: Panepistemio Thessalias Tmema Iatrikes, GREECE

## Abstract

**Background:**

Cognitive impairment is a common non-motor symptom of Parkinson’s disease (PD), yet its underlying mechanisms remain poorly understood. *Apolipoprotein E4 (APOE4)*, a genetic risk factor of Alzheimer’s Disease, has been associated with PD-related cognitive impairment. However, findings are inconsistent, highlighting the need for further investigation. Neuroimaging studies have found gray matter abnormalities, mainly reductions in gray matter volume (GMV) and cortical thickness (CTh), in both cognitively impaired PD patients and *APOE4* carriers. Yet, *APOE4’s* role in these structural changes and their cognitive impact in PD is underexplored.

**Aim:**

This study aimed to determine whether *APOE4* influences early structural brain differences in terms of GMV and CTh in PD prior to the emergence of cognitive dysfunction.

**Methods:**

A total of 51 PD *APOE4* carriers and 120 non-carriers who were cognitively unimpaired from the Parkinson’s Progression Markers Initiative (PPMI) database were included. T1-weighted MRI scans were used to calculate GMV and CTh in regions previously associated with PD-related cognitive impairment, including hippocampal subregions. Cognitive scores assessing global cognition and specific cognitive domains were used to examine associations between regions showing significant GMV or CTh group differences and cognitive performance.

**Results:**

PD *APOE4* carriers showed increased GMV in the left angular gyrus (AnG) and decreased GMV in the left nucleus accumbens (NAcc) compared to non-carriers, though neither survived multiple comparison correction. Left AnG GMV correlated with visuospatial function in both groups but did not remain significant after co-variate adjustment. Left NAcc GMV correlated with visuospatial function and working memory, but only in non-carriers even after co-variate adjustment. No group differences were observed in CTh measures and hippocampal subregion GMVs.

**Conclusions:**

This study suggests that *APOE4* may not influence cognitive function in PD by affecting GMV and CTh. However, longitudinal analyses must confirm these observations.

## Introduction

Parkinson’s Disease (PD) is a complex neurodegenerative disorder that is traditionally defined by clinical motor features of resting tremor, muscle stiffness, impaired coordination, and reduced motor speed [1]. Though motor symptoms are the hallmarks of PD, non-motor symptoms (NMS) also negatively affect patients, substantially decreasing quality of life [2,3]. Cognitive impairment is one of the most common NMS of PD, which can manifest prior, at the time, or decades after diagnosis [1,3]. While research has advanced in exploring underlying mechanisms associated with PD-related cognitive impairment, our understanding and treatment of this non-motor symptom still lags advancements when compared to motor symptoms [2].

*Apolipoprotein E4* (*APOE4*), one of the major genetic risk factors of Alzheimer’s Disease (AD), is proposed to be associated with cognitive impairment in PD [4]. *APOE4* is one of the three alleles (*APOE2* and *APOE3*) of the *APOE* gene, a gene essential for lipid metabolism by transporting cholesterol and other fats in the brain when translated [5]. Evidence show that the *APOE4* allele is more prevalent in PD patients with mild cognitive impairment (PD-MCI) and dementia (PD-D) when compared to cognitively unimpaired healthy controls (HCs) and PD subjects with normal cognition (PD-NC) [4,6,7]. In addition, PD *APOE4* carriers show an increased risk and faster progression to PD-MCI and PD-D, along with greater deficits in cognitive domains such as executive function and memory when compared to non-carriers and/or HCs [4,6]. Considering *APOE4*’s well-established association with AD, *APOE4* has been proposed to exert AD-like neurodegenerative changes that may lead to cognitive changes found in PD patients with *APOE4* [4,5]. This is supported by post-mortem observations of amyloid-beta protein aggregations in those with PD-related cognitive impairment, one of the main AD-protein pathologies, co-existing with Lewy-body pathology, one of the main PD-protein pathologies [4,5]. Despite this evidence, findings are contradictory, where several studies examined no relationship between *APOE4* and cognitive status in PD instead [4], nor with a higher risk of developing PD-D in the future [8–12]. Therefore, it is essential to understand the role of *APOE4* in PD cognition to mitigate these inconsistencies.

Structural magnetic resonance imaging (MRI) has been widely used for the identification of neuroanatomical changes associated with PD-related cognitive impairment [13]. In recent years, gray matter (GM) abnormalities have been found prevalent in PD subjects exhibiting cognitive changes [13]. For example, decreases in gray matter volume (GMV) in specific regions of interests (ROIs) essential for cognitive function when compared to those without cognitive impairment or HCs have been numerously found, including in the frontal and temporal regions, hippocampus, insula, and amygdala [13]. Reduction in cortical thickness (CTh) in cognitive-related regions have also been examined in PD subjects with cognitive impairment, including in the frontal areas and parietal-temporal cortices [13]. Although these abnormalities are prevalent in *APOE4* carriers independent of PD and PD-related cognitive impairment when compared to non-carriers and HCs as well [14,15], limited studies have investigated the effect of *APOE4* on these specific structural MRI measures and explored their potential relationship with cognitive function in PD. Nicoletti and colleagues [16] analyzed these associations in terms of GMV, finding no association between *APOE4* and whole brain GMV in PD patients with and without dementia, while Chung and colleagues [17] investigated whether PD *APOE4* carriers and PD patients with an *APOE3/APOE3* genotype who did not differ in cognitive function showed differences in regional GMVs. Conversely, Sakurai and colleagues [18] found in a cohort of subjects with neurodegenerative diseases, including those with PD, that *APOE4* carriers with slow gait had significantly smaller cerebral GMV that correlated to worsened cognition when compared to non-carriers with normal gait. Only two studies analyzed the potential effect of *APOE4* on CTh in PD subjects [19,20]. One study by Rane and colleagues [20] found that PD *APOE4* carriers with amnestic MCI (a subtype of MCI) had reduced CTh in the middle temporal gyrus when analyzing the default mode network, a critical network known to play a role in attention and memory [21]. Another study by Sampedro and colleagues [19] analyzed that cognitively healthy PD patients with *APOE4* and a specific Brain-Derived Neurotrophic Factor (BDNF) genotype had greater cortical thinning in posterior cortical regions relative to HCs. Thus, it may be worthwhile to further investigate the influence of *APOE4* on gray matter structure in PD to better understand its potential contribution to PD-related cognitive dysfunction.

Notably, cognitively normal *APOE4* carriers show CTh and GMV changes in regions previously associated with cognitive function [22–29]. Likewise, longitudinal MRI studies have also shown that PD subjects who were cognitively normal at baseline but later on developed cognitive impairment already exhibited cortical thinning and GMV reductions at baseline, including in temporal, parietal, occipital, and anterior cingulate regions [30–33]. Additional research studies have identified that baseline GMV reductions in regions such as the nucleus accumbens (NAcc), thalamus, and hippocampal subregions predicted conversion from normal cognition to MCI in PD subjects [34,35]. Collectively, these findings indicate that structural alterations in terms of GMV and CTh can be detected prior to measurable cognitive impairment in both PD subjects and *APOE4* carriers. As a result, this study aimed to investigate whether *APOE4* influences changes in brain structure within regions implicated in cognition in PD, specifically gray matter alterations measured by GMV and CTh, using data from the Parkinson’s Progression Markers Initiative (PPMI). The primary objective was to determine whether *APOE4* status affects GMV and CTh specifically prior to the onset of cognitive dysfunction using a cognitively healthy PD cohort. To achieve this, analysis focused on investigating the GMV and CTh of specific ROIs selected due to their central role in cognition in PD, and exploratory ROIs, which included hippocampal subregions [13]. By examining a cognitively unimpaired PD cohort, this study sought to clarify whether *APOE4* is associated with adverse presymptomatic brain changes that manifest prior to PD-related cognitive impairment, potentially aiding risk identification and supporting early intervention of this non-motor symptom.

## Materials and methods

### Participants

Data used in the preparation of this article was obtained on [2024-2-11] from the Parkinson’s Progression Markers Initiative (PPMI) database (www.ppmi-info.org/access-data-specimens/download-data), RRID:SCR_006431. For up-to-date information on the study, visit www.ppmi-info.org. In accordance with the PPMI data use agreement, the authors of this article did not have access to any information that could identify individual participants during or after data collection, as all PPMI data are de-identified to protect participant privacy. The PPMI is a longitudinal, multi-center observational study aiming to identify PD progression biomarkers to better understand PD etiology and explore potential therapeutic targets [36,37]. Participant enrollment for the Parkinson’s Progression Initiative began in June 2010 and all PD subjects were required to be at least 30 years of age at the time of enrollment [36,37]. The PPMI study was conducted in full accordance with the Declaration of Helsinki and Good Clinical Practice (GCP) guidelines. All participating PPMI clinical sites received ethical approval from their local Institutional Review Boards (IRBs) or Independent Ethics Committee (IECs) on human experimentation before study commencement and received informed written consent from all participants in the study.

Two study groups with baseline measures were examined: PD *APOE4* carriers and non-carriers. All PD participants in the PPMI were considered to be eligible if they had a clinical diagnosis of PD and a positive dopamine transporter (DAT) SPECT scan to confirm DAT binding deficits that represent dopaminergic degeneration, a hallmark of PD pathology [36]. At the time of enrolment, PD subjects were required to be untreated with PD medications, within two years of diagnosis, and have an asymmetric resting tremor or bradykinesia [36]. Baseline measurements represented when the participant formally entered the PPMI study, indicating early clinical stage of the disease. All subjects underwent an MRI scan at baseline [36]. Along with the exclusion criteria of the PPMI, subjects were further excluded based on our own inclusion criteria. Both PD *APOE4* carriers and non-carriers must have; 1) a pre-processed baseline T1-weighted MRI scan and 2) a Montreal Cognitive Assessment (MoCA) score at baseline to measure cognitive function. PD *APOE4* carriers must have at least 1 *APOE4* allele and non-carriers must have 0. Early measures of the MoCA were conducted during screening visit that occurred approximately 60 calendar days before baseline measures were collected (https://www.ppmi-info.org/sites/default/files/docs/PPMI%20Data%20User%20Guide.pdf). Thus, MoCA scores from screening visit were used as a proxy for baseline measures. Alternatively, MoCA scores closest to the date of baseline MRI scan acquisition were used for analysis. All in all, 52 PD *APOE4* carriers and 123 non-carriers met the inclusion criteria prior to quality control of their processed MRI scans. Following quality control, 4 subjects were excluded due to poor imaging quality and motion artefacts, resulting in a final sample of 51 PD *APOE4* carriers and 120 non-carriers for analysis. All T1-weighted scans were obtained in approved scanners of PPMI centers that abided by imaging center compliances based on the PPMI MRI technical operations manual (https://www.ppmi-info.org/sites/default/files/docs/archives/PPMI2.0_MRI_TOM_Final_FullyExecuted_v2.0_20200807.pdf) and acquired using SIEMENS scanners in sagittal plane at a field strength of 3.0 Tesla.

Demographic characteristics analyzed included age at the time of baseline MRI scan, sex, years of education during screening time, and *APOE4* genotype. Genetic status of other genes (*LRRK2* and *GBA*) was also acquired to potentially account for genetic interactions with *APOE4*. Clinical assessments analyzed included disease duration, defined in our study as the number of months from the time of PD diagnosis to when the baseline MRI scan was acquired, the Movement Disorder Society-Unified Parkinson Disease Ration Scale (MDS-UPDRS) Part III (off medication) to measure parkinsonian severity based on severity of motor symptoms, and MoCA scores. Higher MDS-UPDRS Part III scores indicate greater motor symptom severity and a MoCA score of less than 26 was used as a cut off to indicate cognitive impairment, consistent with a threshold used to identify MCI [38,39]. Additional cognitive testing scores available for all subjects were acquired, considering that PD-related cognitive impairment involves deficits in specific cognitive domains [2]. This included the: Corrected Symbol Digits Modalities (SDM) T-scores (processing speed and attention); Corrected Semantic (Animal) Fluency T-scores (executive function and working memory); Corrected Letter Number Sequencing (LNS) scaled scores (executive function and working memory);Hopkins Verbal Learning Test (HVLT) – Revised T-scores (memory (immediate recall, delayed free recall, retention, and recognition)); and Corrected Benton Judgement of Line Orientation (JOLO) −15 item version (visuospatial function) scaled scores [40]. Published norms were applied on the clinical assessments, including cognitive tests [36]. No data was missing for age, years of education, MoCA scores, sex, and *LRRK2* status; however, 4 subjects were missing data for MDS-UPDRS Part III scores, 1 subject was missing data for *GBA* status, and 2 subjects had missing data for all other cognitive tests. These subjects were not included in the analyses of group differences for these clinical assessments. However, such subjects were still included in the analysis of group differences of clinical and demographical characteristics where they had data.

### Structural MRI processing

Processing of the MRI scans to acquire GMV and CTh measures were performed using FreeSurfer imaging analysis suite, which is documented and freely available for download online (Version 7.1; http://surfer.nmr.mgh.harvard.edu). Processing involved various steps using the “recon-all” function. Briefly, as outlined on the Freesurfer methods citation webpage (https://surfer.nmr.mgh.harvard.edu/fswiki/FreeSurferMethodsCitation), this includes motion correction, averaging multiple volumetric T1-weighted images, brain tissue removal, automated Talariach transformation, subcortical white matter (WM) and deep gray matter volumetric structure segmentation, intensity normalization, GM and WM boundary tessellation, automated topology correction, and surface deformation following intensity gradients to place the GM/WM and GM/cerebrospinal fluid (CSF) borders. Various deformable steps are then performed, including surface inflation, registration of the cortical models to a spherical atlas, parcellation of the cerebral cortex into units with respect to gyral and sulcal structure, and creating surface data, including maps of curvature and sulcal depth. Using this method, CTh was calculated as the closest distance from the GM/WM boundary to the GM/CSF boundary at each vertex on the tessellated surface. Quality control of FreeSurfer’s processing steps were then performed using visual inspection for each scan using FreeSurfer’s Freeview. Pial surface, segmentation, and intensity normalization errors were manually corrected using the subject’s brain or white matter mask.

Cortical ROIs were parcellated from the Desikan-Kiliany (DSK) atlas, and subcortical ROIs were parcellated from FreeSurfer’s automated subcortical structure segmentation. Subcortical ROIs were analyzed using GMV only, as CTh measures are not applicable for deep GM brain structures in FreeSurfer. ROI analyses were conducted in 7 specific regions of interest previously associated with PD-related cognitive impairment, including the biltateral insula, hippocampus, superior frontal gyrus, superior temporal gyrus, inferior parietal cortex (henceforth “angular gyrus”), rostral middle frontal cortex (henceforth “dorsolateral prefrontal cortex (DLPFC)”), and supramarginal gyrus. These regions were chosen based on findings from multiple meta-analyses observing reduced structural or functional changes in those with PD-related cognitive impairment [13,41–45]. Six exploratory ROIs, including the bilateral caudal anterior cingulate (henceforth “anterior cingulate”), thalamus, amygdala, caudate, nucleus accumbens, and entorhinal cortex were also examined. Selection was based on multiple structural MRI studies reporting gray matter atrophy in these areas amongst those with PD-related cognitive impairment, though to a lesser extent than the specific regions selected as previously stated [13]. Bilateral hippocampal subregion GMV (Cornu Ammonis (CA) 1, CA3, CA4, parasubiculum, presubiculum, subiculum, Molecular and Granule Cell Layers of the Dentate Gyrus (GC-ML-DG), Molecular Layer of the Dentate Gyrus (Molecular Layer), Hippocampus-Amygdala Transition Area (HATA), fimbria, hippocampal tail, and hippocampal fissure) were extracted using Freesurfer’s hippocampal subfields tool with Head, Body, Tail (HBT) segmentation [46]. For subregions subdivided into head and body, these values were summed to obtain total subregion GMV, following FreeSurfer’s v6.0 segmentation approach [46].

### Statistical analyses

Statistical analyses were performed using RStudio Version 2024.04.2 + 764 (PBS., Boston, MA). Group differences in clinical and demographical characteristics were analyzed using independent sample t-tests or Mann-Whitney U tests, depending on whether continuous variables were normally distributed. Chi-square or Fisher’s exact tests were used for categorical variables, depending on expected cell counts. Group differences in GMV and CTh across ROIs were first examined using independent t-tests and analysis of covariance (ANCOVA) between PD *APOE4* carriers and non-carriers to examine both unadjusted and co-variate adjusted outcomes, respectively. Co-variates included age, sex, and disease duration. Total Intracranial volume (TIV) was additionally added as a co-variate in GMV analyses to correct for head size. Subsequently, Pearson correlations were then performed to examine the relationship between cognitive scores and ROIs that showed significant group differences in GMV and CTh after co-variate adjustment. For any significant correlations, linear regression models were conducted to adjust for covariates (age, sex, disease duration, and TIV for GMV) if needed. Statistical significance was initially defined as p < 0.05, uncorrected. Bonferroni correction for multiple comparisons was applied as appropriate.

## Results

### Demographic and clinical characteristics

Demographic and clinical characteristics are summarized in Table 1. PD *APOE4* carriers were significantly younger (t_94.77_ = 2.763, *p* = 0.007) and had a shorter disease duration than PD *APOE4* non-carriers (W = 3684, *p* = 0.034). Thus, age and disease duration were used as co-variates in subsequent analyses. No significant group differences were found for sex (χ = 1.138, *p* = 0.286) and TIV (t_87.86_ = −0.401, *p* = 0.690). However, both variables were included as co-variates for subsequent analyses to control for *APOE4*’s potential sex-dependent effect [47] and to correct for individual differences in head size, respectively. There were no group differences in years of education, *LRRK2* and *GBA* status, MDS-UPDRS Part III scores, and any cognitive measures. Both PD *APOE4* carriers and non-carriers exhibited mild motor impairment, as indicated by their mean MDS-UPDRS Part III scores (20.176 ± 9.014 and 20.982 ± 9.415, respectively) and were cognitively normal based on their MoCA scores (> 26).

**Table 1:** Demographic and clinical characteristics of PD *APOE4* carriers and non-carriers.

	PD *APOE4* carriers	PD *APOE4* non-carriers	P-value (p < 0.05)
Sample Size (n)	51	120	–
*APOE4* genotype			
*E4/E4*	6	–	–
*E3/E4*	41	–	–
*E2/E4*	4	–	–
*E2/E2*	–	2	–
*E3/E3*	–	100	–
*E2/E3*	–	18	–
**Age (Years)**	**58.745 ± 9.110**	**62.958 ± 9.152**	**0.007**
Years of Education	15.804 ± 2.863	15.350 ± 3.091	0.357
**Disease Duration (Months)** ^**a**^	**4.000 [5.500]**	**6.000 [11.250]**	**0.034**
MDS-UPDRS III	20.176 ± 9.014	20.982 ± 9.415	0.601
eTIV (mm^3^)	1522599.86 ± 157216.90	1512300.39 ± 14191.30	0.690
Sex			0.286
Females	16	48	
Males	35	72	
*LRRK2* Status^b^			0.398
Yes	3	13	
No	48	107	
*GBA* Status^b^			1.00
Yes	4	9	
No	46	111	
MoCA Scores	27.745 ± 2.261	27.458 ± 1.982	0.434
LNS Scaled Scores	12.353 ± 2.521	11.542 ± 2.775	0.066
SDM T-scores	46.282 ± 10.923	45.322 ± 8.495	0.578
SF (Animals) T-scores	52.411 ± 10.220	51.288 ± 9.667	0.507
HVLT-R Total Recall T-scores	45.157 ± 13.267	47.245 ± 10.959	0.326
HVLT-R Delayed Recall T-scores	45.490 ± 12.602	45.610 ± 12.023	0.954
HVLT-R Recognition T-scores	45.843 ± 12.667	44.568 ± 12.356	0.547
HVLT-R Retention T-scores^a^	48.000 [14.500]	49.500 [14.750]	0.567
JOLO Scaled Scores	12.097 ± 3.222	12.014 ± 2.759	0.872

Data presented as mean ± standard deviation for continuous variables and counts for categorical variables. T-scores have a mean of 50 with a standard deviation of 10. Scaled scores have a mean of 10 with a standard deviation of 3 [48]. Abbreviations: MDS-UPDRS III, Movement Disorder Society-Unified Parkinson’s Ration Scale Part 3 (off medication); MoCA, Montreal Cognitive Assessment; SDM, Symbol Digits Modalities; LNS, Letter Number Sequencing; SF, Semantic Fluency; HVLT-R, Hopkins Verbal Learning Test- Revised; JOLO, Benton Judgement of Line Orientation – 15 item version; eTIV, estimated Total Intracranial Volume.

^a^Data presented as median [Interquartile-Range] due to use of non-parametric test (Mann-Whitney U Test) for continuous variables that are not normally distributed.

^b^Group difference in categorical variable was analyzed using Fisher’s Exact Tests due to cell counts being less than 5.

### GMV analyses

**Fig 1:**
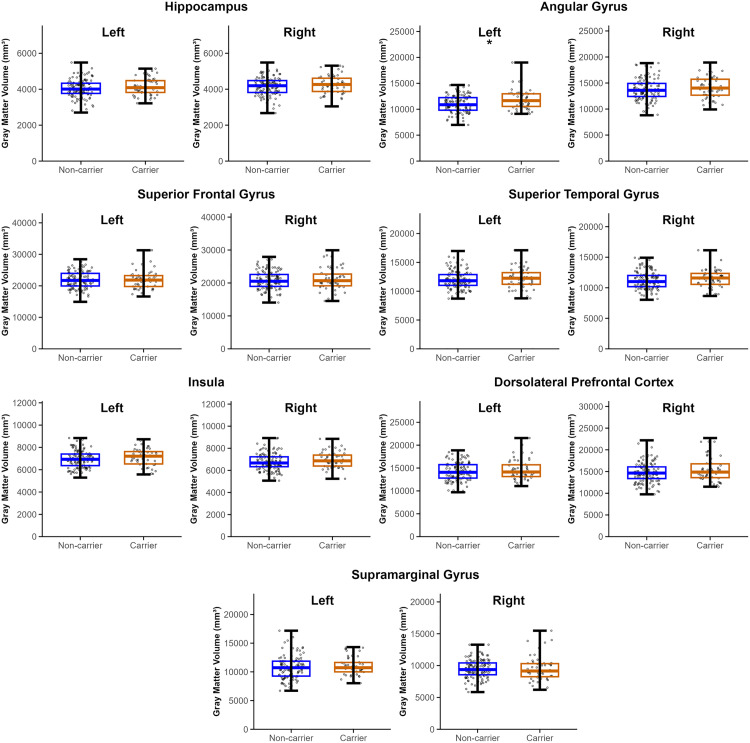
Mean gray matter volume (GMV) of 7 specific regions of interest for PD *APOE4* carriers and PD *APOE4* non-carriers. A significant increase in GMV was observed in the left angular gyrus of PD *APOE4* carriers when compared to PD *APOE4* non-carriers after adjusting for age, sex, disease duration, and TIV (*p < 0.05, uncorrected); however, this did not survive Bonferroni correction for multiple comparisons (p > 0.004). Bars on boxplots represent minimum and maximum GMV values.

For the 7 specific ROIs, only the left angular gyrus (AnG) showed a significant difference in GMV between the two study groups for both unadjusted analyses (t_83.42_ = −2.568, *p* = 0.012) and in ANCOVA models adjusted for age, sex, disease duration, and TIV (*F*_*1,165*_ = 6.702, *p =* 0.010) (Fig 1, S1 Table), with PD *APOE4* carriers exhibiting higher GMV than non-carriers. However, no GMV differences in any of the specific ROIs survived Bonferroni correction (p > 0.04), therefore follow up analyses are considered exploratory.

Exploratory Pearson correlations showed that the left AnG GMV was significantly correlated with JOLO scores with no co-variate adjustment in the whole PD cohort (r_167_ = 0.267; *p* < 0.001), as well as in PD *APOE4* carriers (r_49_ = 0.323, *p =* 0.021) and non-carriers (r_116_ = 0.239, *p =* 0.009) (S2 Table). Exploratory linear regression models adjusted for age, sex, disease duration, and TIV were subsequently performed for all three correlations, and none remained significant (p > 0.05, uncorrected) (S3 Table).

**Fig 2:**
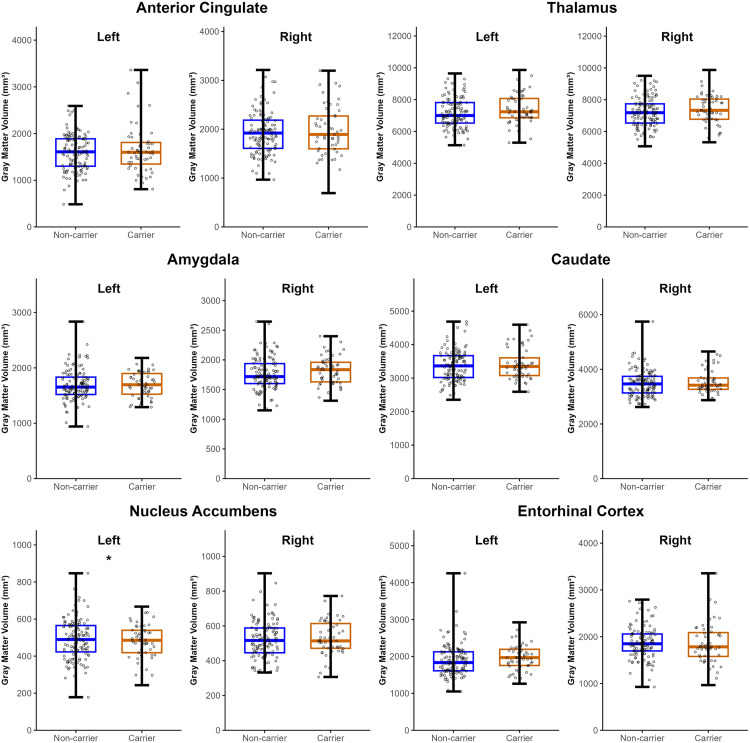
Mean gray matter volume (GMV) of exploratory regions of interests for PD *APOE4* carriers and PD *APOE4* non-carriers. A significant decrease in GMV was observed in the left nucleus accumbens of PD *APOE4* carriers when compared to PD *APOE4* non-carriers after adjusting for age, sex, disease duration, and TIV (*p < 0.05, uncorrected); however, this did not survive Bonferroni correction for multiple comparisons (p > 0.004). Bars on boxplots represent minimum and maximum values.

For the 6 exploratory ROIs, group differences did not reach significant in any of the ROIs in the unadjusted analyses (p > 0.05, uncorrected). ANCOVA models then revealed that PD *APOE4* carriers had significantly lower GMV in the left nucleus accumbens (NAcc) when adjusting for age, sex, disease duration, and TIV compared to non-carriers (F_1,165_ = 6.034, *p =* 0.015) (Fig 2, S4 Table). However, this did not survive Bonferroni correction (p > 0.004), and therefore follow-up analyses are also considered exploratory.

In exploratory correlations, it revealed that the left NAcc GMV was significantly correlated with JOLO and LNS scores when not accounting for co-variates in the whole PD cohort (JOLO: r_167_ = 0.230, *p* = 0.003; LNS: r_167_ = 0.200, *p =* 0.009). These correlations were also examined in PD *APOE4* non-carriers (JOLO: r_116_ = 0.271, p *=* 0.003; LNS:r_116_ = 0.219, *p =* 0.017) but not carriers (JOLO: r_49_ = 0.144,*p =* 0.315; LNS:r_49_ = 0.171, *p =* 0.231 (S2 Table)). Exploratory regression models adjusted for age, sex, disease duration and TIV were subsequently performed for the significant correlations (See S5 and S6 Table). The association between left NAcc GMV and LNS remained significant only in the non-carriers (F _(5,112)_ =2.538, *p =* 0.033), however this did not survive Bonferroni correction (p > 0.013) (S6 Table).

### CTh analyses

**Fig 3:**
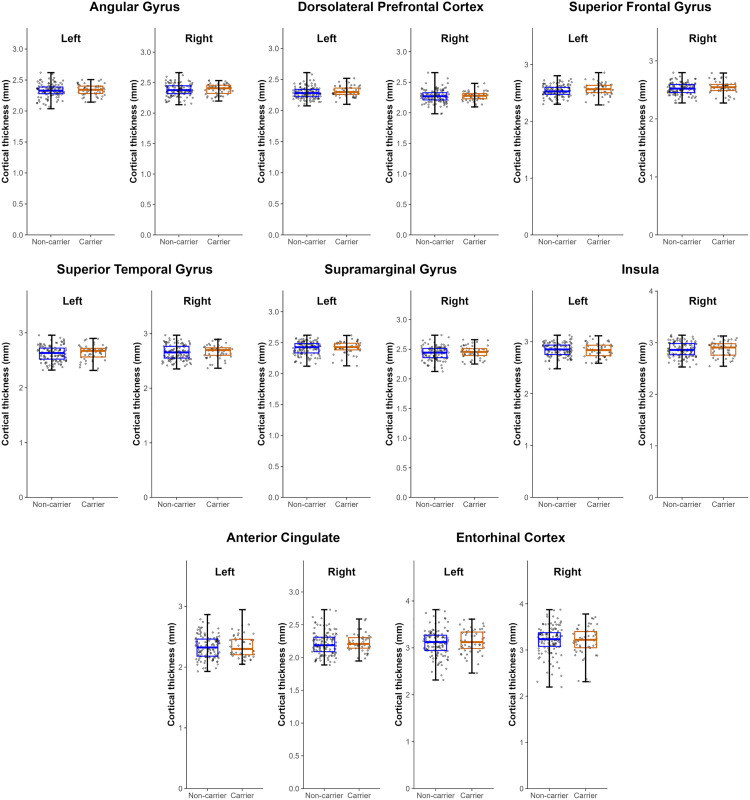
Mean Cortical Thickness (CTh) of specific and exploratory regions of interest for PD *APOE4* carriers and PD *APOE4* non-carriers. No significant CTh differences were found between the two groups for both specific and exploratory regions after adjusting for age, sex, and disease duration (p > 0.05, uncorrected). Bars on boxplots represent minimum and maximum values.

Unadjusted and adjusted analyses revealed no significant group differences in CTh in any of the specific and exploratory ROIs (Fig 3, S7 Table). Therefore, no further analyses were done.

### Hippocampal subregion GMV analyses

**Fig 4:**
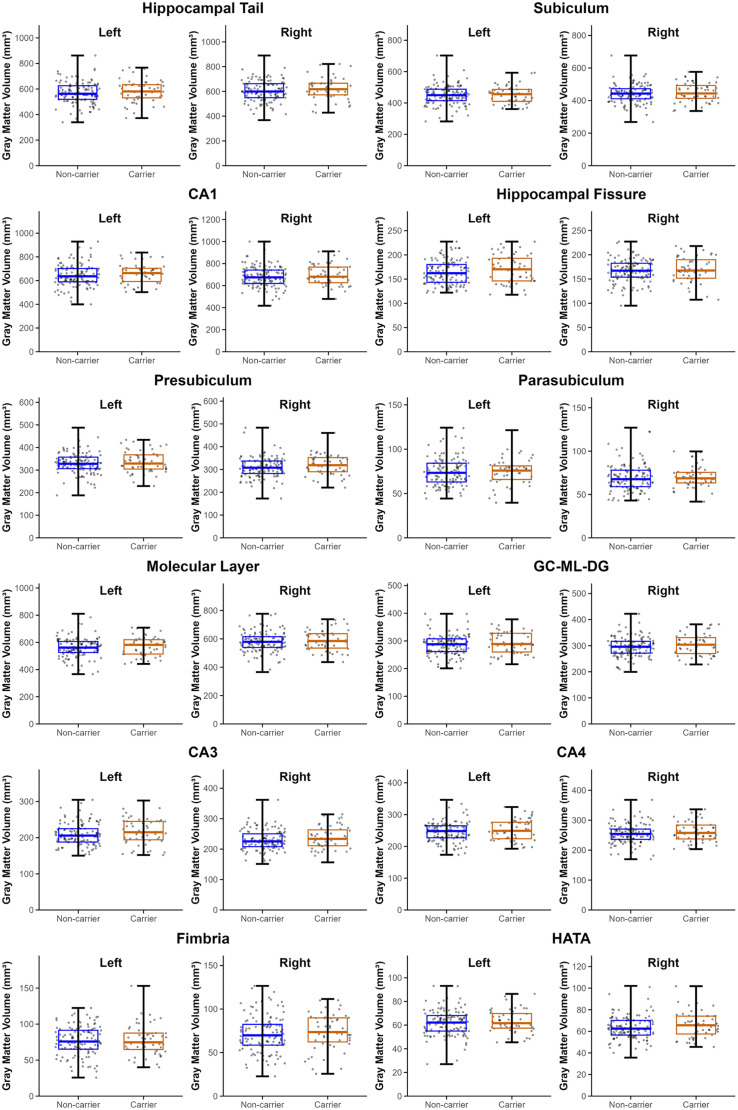
Mean gray matter volume (GMV) of bilateral hippocampal subregions for PD *APOE4* carriers and PD *APOE4* non-carriers. No significant GMV differences in the subregions were found between the two groups after adjusting for age, sex, disease duration, and TIV (p > 0.05, uncorrected). Abbreviations: CA, Cornu Ammonis; HATA, Hippocampus-Amygdala Transition Area; Molecular Layer, Molecular layer of the dentate gyrus; GC-ML-DG, Granule cell and molecular layer of the dentate gyrus. Bars on boxplots represent minimum and maximum values.

Unadjusted and adjusted analyses revealed no significant group differences in GMV of all hippocampal subregions (Fig 4, S8 Table). Therefore, no further analyses were conducted.

## Discussion

This study found no robust evidence that *APOE4* plays a role in PD by influencing structural brain changes associated with cognitive dysfunction, measured via GMV and CTh in cognitively healthy individuals. No group differences in CTh were observed across specific and exploratory ROIs, nor were there differences in hippocampal subregion GMVs. Though PD *APOE4* carriers showed greater GMV in the left AnG and lower GMV in the left NAcc compared to non-carriers after co-variate adjustment, these did not survive correction for multiple comparisons and were not associated with cognitive performance, indicating limited statistical support.

It is important to reiterate that our PD cohort did not meet the clinical criteria for cognitive impairment as defined by MoCA scores for PD-MCI. Previous studies have identified that *APOE4* carriers classified to be cognitively normal exhibit GMV changes in regions essential for cognitive function, such as in the hippocampus and insula, suggesting that these structural alterations may precede the development of cognitive impairment [22,24,25]. For example, Haller and colleagues [25] found that cognitively normal *APOE4* carriers showed gray matter loss in the posterior cingulate at baseline when compared to those with *APOE2* and *APOE3*, and later exhibited cognitive decline during a 18-month follow-up. Various studies have also observed that cognitively normal individuals with *APOE4* who exhibit higher baseline amyloid-beta accumulations, the hallmark AD protein pathology central to neurodegeneration and has been linked to gray matter alterations [22,49,50], demonstrate faster rates of cognitive decline in various cognitive processes over time [51–53]. However, in the present study, *APOE4* was not associated with significant changes in GMV within a cognitively normal cohort of early-stage PD subjects. This suggests that *APOE4*-related effects on gray matter may not be evident specifically during the initial phases of PD when cognitive function is intact. This interpretation is supported with previous reviews that have proposed that *APOE4’*s effect on PD-related cognitive impairment could be time-dependent, with having a pronounced effect later in the disease just like AD [4]. Since PD-MCI generally occurs later in PD, with about 25% of PD patients experiencing it at the time of diagnosis and about 40–50% after a 5 year follow up [2], it is possible that *APOE4*’s influence on GMV and CTh may have not manifested yet in our study. Correspondingly, recent studies have also reported no significant effects of *APOE4* on cognitive performance in PD subjects during the early stages of the disease, which is consistent with the absence of cognitive differences between the PD *APOE4* carriers and non-carriers in our study [54]. Thus, our findings may reflect a disease stage-dependent role of *APOE4* in PD, where its effects on gray matter as well as its association with cognition may emerge later during PD progression and when cognitive dysfunction becomes more prevalent.

Although the analyses in the present study did not reach statistical significance, such findings should be interpreted with caution. The use of Bonferroni correction for our analyses to control for multiple comparisons is known to be conservative and may have increased the risk of Type II errors. Thus, this may have masked small, meaningful true effects, contributing to the insignificant results for the GMV analyses once corrected. Such findings should therefore be interpreted as exploratory and alternative correction methods in future studies should be considered. In addition, the lack of structural differences observed between the two study groups may be attributable to the conservative nature of the sample. As both groups were cognitively normal, any pathological changes associated with *APOE4* that may affect cognition could have not yet occurred, limiting our ability to detect group differences. Taking this all into consideration, the uncorrected GMV differences we observed in the left AnG and NAcc may indicate early and subtle structural changes prior to symptom development associated with *APOE4* in PD. These findings can guide future longitudinal investigations. Given the availability of longitudinal data for PD *APOE4* carriers and non-carriers from baseline to 1-,2-, and 4- year follow ups under consistent inclusion and exclusion criteria of the present study, these current findings may provide a basis for analysis aimed at tracking *APOE4*’s effect on gray matter changes and its associations with cognitive trajectories overtime. Here, we discuss the implications of atrophy within these regions and the impact on cognitive function.

The AnG is proposed to integrate multisensory information and has shown atrophy in those with PD-MCI and in individuals with *APOE4* [13,42]. Our PD *APOE4* cohort showed an increased GMV of the left AnG, rather than a reduction. Increased GMV may be due to increased neuroinflammation as a compensatory response to neurodegeneration, especially given *APOE4’*s well-established role in promoting microglial activation [5,55]. Interestingly, Femminella and colleagues [56] observed microglial activation to be associated with higher GMV in individuals with early MCI. Thus, increased GMV in the left AnG of PD *APOE4* carriers may represent an early glial-mediate response prior to onset of cognitive changes. However, we found no correlation between left AnG GMV and visuospatial ability (JOLO scores) following co-variate adjustment. Therefore, we cannot conclude that the GMV change in this region potentially due to *APOE4* in PD patients has a greater influence on cognitive function than age, sex, disease duration, or TIV.

The NAcc is part of the limbic system and networks that underlie cognitive processes, including working memory, spatial and contextual processing, and attention [57]. Reduced GMV in this region has been linked to cognitive deficits in PD patients, suggesting that our results showing degeneration within this region (uncorrected) in PD *APOE4* carriers align with previous reports of PD-related atrophy associated with cognitive impairment [13]. However, we only saw a relationship between NAcc GMV with visuospatial ability (JOLO scores) as well as memory and executive function (LNS scores) in PD *APOE4* non-carriers. Thus, this suggests that *APOE4* may not be driving changes in NAcc GMV of the PD brain related to cognitive function in our study. Overall, since both groups generally performed well in most cognitive assessments, including the LNS and JOLO, it is unlikely to see strong, robust correlations between any cognitive scores and the specific MRI measures. A more diverse cohort with varying cognitive statuses may be worthwhile to further understand the relationship between *APOE4*, GMV and CTh, and cognitive function in PD.

In addition to observing subjects during the early stages of the disease, we also outline other study limitations. The age differences between our study groups required to include age as a co-variate for our analyses. However, this adjustment may not fully account for potential age-related brain changes associated with *APOE4*, which may only become detectable in later adulthood. Aging is a well-established predictor of PD-D development and has been associated with greater GMV and CTh atrophy over time, with elderly individuals showing greater atrophy than those younger [1,2,58,59]. Interestingly, *APOE4* has been proposed to exert age-dependent effects by conferring cognitive advantages in younger subjects, a concept referred to as the “antagonistic pleiotropy hypothesis” [60]. This concept proposes that some genes have different impacts during life stages, with beneficial effects during early life but detrimental outcomes in later age [60]. For *APOE4*, it has been examined that younger *APOE4* carriers outperform non-carriers cognitively, and that *APOE4* is then associated with cognitive decline in old age [60]. Though speculative, this may partly explain the absence of structural brain differences between our study groups since our carriers were significantly younger, possibly contributing to their preserved cognitive function. Having a smaller sample size of PD *APOE4* carriers compared to non-carriers is also a limitation. Since our sample of carriers is almost half of the non-carriers, our results may not be as representative of the general PD *APOE4* carrier population. Future studies should aim to have a balanced design for the study groups if feasible to garner more statistical power. Further, it should be acknowledged that the present study relied on data only from the PPMI. While this dataset has been increasingly used in PD research, analysis based on a single cohort may be subjected to cohort-specific biases, including specific recruitment criteria. Thus, future studies that examine our objectives in other independent cohorts may increase generalizability, which is essential in validating our findings to the wider PD population. Lastly, it should be considered that some brain regions examined were based on proxy brain areas. This is since these regions are not well-defined in the atlas used, and these proxies were chosen due to anatomically containing the ROI and have been used in previous literature to represent such region [61–63]. A more streamlined approach that directly targets the regions in the future may be worthwhile to ensure accuracy and reliability of analyses.

## Conclusion

This study revealed that PD subjects with *APOE4* do not exhibit GMV and CTh changes that may reflect changes in cognitive function. Though PD *APOE4* carriers were observed to have GMV changes in the left AnG and NAcc when compared to non-carriers, these changes did not remain significant after statistical correction, ultimately lacking robustness. These findings overall suggest that *APOE4* may not affect the GMV and CTh in cognitively unimpaired, PD individuals, and therefore, may potentially not serve as an early biomarker for PD-related cognitive impairment. Future studies are warranted to investigate *APOE4’*s effect longitudinally to confirm whether these observations persists and if it exerts a time-dependent influence instead.

## Supporting information

S1 Table Adjusted group comparisons of gray matter volume across specific regions between PD *APOE4* carriers and non-carriers. Data presented as adjusted mean (Standard Error) in mm^3^. All statistical tests were adjusted for age, sex, disease duration, and eTIV as co-variates. Abbreviations: Lh, left hemisphere; Rh, right hemisphere; eTIV, estimated total intracranial volume; ANG, Angular Gyrus; DLPFC, Dorsolateral Prefrontal Cortex; HPC, Hippocampus; INS, Insula; SFG, Superior Frontal Gyrus; STG, Superior Temporal Gyrus; SMG, Supramarginal Gyrus. ^a^ P-values are reported as uncorrected, with a p-value threshold of 0.05. No p-values are emphasized, as none survived Bonferroni correction for multiple comparison correction (p > 0.004).
(DOCX)

S2 Table Correlation analyses between cognitive scores and gray matter volume of left angular gyrus and left nucleus accumbens in the whole PD cohort and subgroup analyses. Analysis was first conducted in the whole cohort to identify significant correlations. Subgroup analyses (PD *APOE4* carriers and PD *APOE4* non-carriers) were then performed for cognitive tests that showed significant correlations in the whole group analyses. Significant correlations are bolded. Horizontal lines indicate the transition from whole cohort results to subgroup analyses. Abbrevations: Lh, left hemisphere; ANG, Angular Gyrus; NAcc, Nucleus Accumbens; JOLO, Benton Judgement of Line Orientation – 15 item version; HVLT-R, Hopkins Verbal Learning Test- Revised; SF, Semantic Fluency; SDM, Symbol Digits Modalities Test; LNS, Letter Number Sequencing; Carriers, PD *APOE4* carriers; Non-carriers, PD *APOE4* non-carriers. ^a^ P-values are reported as uncorrected, with a p-value threshold of 0.05 (statistical significance in bold).
(DOCX)

S3 Table Regression models adjusted for covariates to examine the association between left angular gyrus gray matter volume and JOLO scores for whole PD cohort and subgroup analyses. Analysis of significant associations between left AnG GMV and JOLO scores when adjusting for age, sex, disease duration, and eTIV. Significant associations after co-variate adjustment are bolded. Abbreviations: Lh, left hemisphere; GMV, gray matter volume; eTIV, estimated total intracranial volume; AnG; Angular Gyrus; JOLO, Benton Judgement of Line Orientation – 15 item version; CI, Confidence Interval; β, Beta Coefficient; SE, Standard Error. ^a^ P-values are reported as uncorrected, with a p-value threshold of 0.05 (statistical significance in bold).
(DOCX)

S4 Table Adjusted group comparisons of gray matter volume across exploratory regions between PD *APOE4* carriers and non-carriers. Data presented as adjusted mean (Standard Error) in mm^3^. All statistical tests were adjusted for age, sex, disease duration, and eTIV as co-variates. Abbrevations: Lh, left hemisphere; Rh, right hemisphere; eTIV, estimated total intracranial volume; ACC, Anterior Cingulate; Thal, Thalamus; Amyg, Amygdala; Caud, Caudate; NAcc, Nucleus Accumbens; EC, Entorhinal Cortex. ^a^ P-values are reported as uncorrected, with a p-value threshold of 0.05. No p-values are emphasized, as none survived Bonferroni correction for multiple comparison correction (p > 0.004).
(DOCX)

S5 Table Regression models adjusted for covariates to examine the association between the left nucleus accumbens gray matter volume and JOLO scores for whole PD cohort and subgroup analyses. Analysis of significant associations between left NAcc GMV and JOLO scores when adjusting for age, sex, disease duration, and eTIV. Significant associations after co-variate adjustment are bolded. Abbreviations: Lh, left hemisphere; GMV, gray matter volume; eTIV, estimated total intracranial volume; NAcc, Nucleus Accumbens; JOLO, Benton Judgement of Line Orientation – 15 item version; CI, Confidence Interval; β, Beta Coefficient; SE, Standard Error. ^a^ P-values are reported as uncorrected, with a p-value threshold of 0.05 (statistical significance in bold).
(DOCX)

S6 Table Regression models adjusted for covariates to examine the association between the left nucleus accumbens gray matter volume and LNS scores for whole PD cohort and subgroup analyses. Analysis of significant associations between left NAcc GMV and LNS scores when adjusting for age, sex, disease duration, and eTIV. Significant associations after co-variate adjustment are bolded. Abbreviations: Lh, left hemisphere; eTIV, estimated total intracranial volume; NAcc, Nucleus Accumbens; LNS, Letter Number Sequencing Scores; CI, Confidence Interval; β, Beta Coefficient; SE, Standard Error. ^a^ P-values are reported as uncorrected, with a p-value threshold of 0.05 (statistical significance in bold).
(DOCX)

S7 Table Adjusted group comparisons of cortical thickness across specific and exploratory regions between PD *APOE4* carriers and non-carriers. Data presented as adjusted mean (Standard Error) in mm. All statistical tests were adjusted for age, sex, and disease duration as co-variates. Bold Horizontal line separates specific regions from exploratory regions. Abbreviations: Lh, left hemisphere; Rh, right hemisphere; noncarriers, PD *APOE4* non-carriers; carriers, PD *APOE4* carriers; ANG, Angular Gyrus; DLPFC, Dorsolateral Prefrontal Cortex; HPC, Hippocampus; INS, Insula; SFG, Superior Frontal Gyrus; STG, Superior Temporal Gyrus; SMG, Supramarginal Gyrus; ACC, Anterior Cingulate; EC, Entorhinal Cortex. ^a^ P-values are reported as uncorrected, with a p-value threshold of 0.05 (statistical significance in bold).
(DOCX)

S8 Table Adjusted group differences of gray matter volume across hippocampal subregions between PD *APOE4* carriers and non-carriers. Data presented as adjusted mean (Standard Error) in mm^3^. All statistical tests were adjusted for age, sex, disease duration, and eTIV as co-variates. Abbreviations: Lh, left hemisphere; Rh, right hemisphere; eTIV, estimated total intracranial volume; Hipp, Hippocampal; noncarriers; PD *APOE4* non-carriers; carriers, PD *APOE4* carriers; CA, Cornu Ammonis; Molecular Layer, Molecular layer of the dentate gyrus; GC-ML-DG, Granule cell and molecular layer of the dentate gyrus. ^a^ P-values are reported as uncorrected, with a p-value threshold of 0.05 (statistical significance in bold).
(DOCX)
