# Supplementary material for: The effect of Apolipoprotein E4 on cognitive function in Parkinson’s disease: A structural MRI study in the PPMI cohort
Source: PLoS One. 2026 Jan 20;21(1):e0341240. doi: 10.1371/journal.pone.0341240 (PMC12818682; doi:10.1371/journal.pone.0341240)
Supplement: S1 Table — Data presented as adjusted mean (Standard Error) in mm3. All statistical tests were adjusted for age, sex, disease duration, and eTIV as co-variates. Abbreviations: Lh, left hemisphere; Rh, right hemisphere; eTIV, estimated total intracranial volume; ANG, Angular Gyrus; DLPFC, Dorsolateral Prefrontal Cortex; HPC, Hippocampus; INS, Insula; SFG, Superior Frontal Gyrus; STG, Superior Temporal Gyrus; SMG, Supramarginal Gyrus. a P-values are reported as uncorrected, with a p-value threshold of 0.05. No p-values are emphasized, as none survived Bonferroni correction for multiple comparison correction (p > 0.004). (DOCX) [file pone.0341240.s001.docx]

**Supplementary Table 1: Adjusted group comparisons of gray matter volume across specific regions between PD *APOE4* carriers and non-carriers.**

| **Region of Interest** | **Adjusted mean (non-carriers)** | **Adjusted mean**  **(carriers)** | **P**  **value**^a^ | **R^2^**  **adjusted** | **η2**  **partial** | **F**  **value** |
| --- | --- | --- | --- | --- | --- | --- |
| Lh HPC | 4069.053 (36.021) | 4082.499(56.430) | 0.839 | 0.381 | 0.000 | 0.041 |
| Lh ANG | 11197.247(129.057) | 11809.774(202.179) | 0.010 | 0.387 | 0.039 | 6.702 |
| Lh DLPFC | 14312.619(132.458) | 14421.299(207.507) | 0.655 | 0.541 | 0.001 | 0.200 |
| Lh SFG | 21931.434(164.04) | 21467.1(256.982) | 0.125 | 0.586 | 0.014 | 2.384 |
| Lh STG | 12067.584(102.351) | 11913.184(160.340) | 0.412 | 0.522 | 0.004 | 0.677 |
| Lh SMG | 10816.942(135.423) | 10731.491(212.151) | 0.731 | 0.388 | 0.001 | 0.118 |
| Lh INS | 6948.523(50.478) | 6989.344(79.077) | 0.660 | 0.486 | 0.001 | 0.195 |
| Rh HPC | 4192.573(36.208) | 4171.768(56.723) | 0.754 | 0.396 | 0.001 | 0.098 |
| Rh ANG | 13850.776(141.254) | 13842.68(221.286) | 0.975 | 0.462 | 0.000 | 0.001 |
| Rh DLPFC | 14748.553(190.346) | 15392.101(298.192) | 0.067 | 0.327 | 0.020 | 3.401 |
| Rh SFG | 21028.558(198.746) | 21105.678(311.351) | 0.833 | 0.496 | 0.000 | 0.045 |
| Rh STG | 11297.708(92.934) | 11278.428(145.589) | 0.910 | 0.525 | 0.000 | 0.013 |
| Rh SMG | 9599.553(118.333) | 9412.84(185.379) | 0.391 | 0.368 | 0.004 | 0.741 |
| Rh INS | 6757.74(50.025) | 6849.309(78.369) | 0.320 | 0.488 | 0.006 | 0.997 |

Data presented as adjusted mean (Standard Error) in mm^3^. All statistical tests were adjusted for

age, sex, disease duration, and eTIV as co-variates. Abbreviations: Lh, left hemisphere; Rh, right hemisphere; eTIV, estimated total intracranial volume; ANG, Angular Gyrus; DLPFC, Dorsolateral Prefrontal Cortex; HPC, Hippocampus; INS, Insula; SFG, Superior Frontal Gyrus; STG, Superior Temporal Gyrus; SMG, Supramarginal Gyrus.

^a^ P-values are reported as uncorrected, with a p-value threshold of 0.05. No p-values are emphasized, as none survived Bonferroni correction for multiple comparison correction (p > 0.004).
