# Supplementary material for: The effect of Apolipoprotein E4 on cognitive function in Parkinson’s disease: A structural MRI study in the PPMI cohort
Source: PLoS One. 2026 Jan 20;21(1):e0341240. doi: 10.1371/journal.pone.0341240 (PMC12818682; doi:10.1371/journal.pone.0341240)
Supplement: S2 Table — Analysis was first conducted in the whole cohort to identify significant correlations. Subgroup analyses (PD APOE4 carriers and PD APOE4 non-carriers) were then performed for cognitive tests that showed significant correlations in the whole group analyses. Significant correlations are bolded. Horizontal lines indicate the transition from whole cohort results to subgroup analyses. Abbrevations: Lh, left hemisphere; ANG, Angular Gyrus; NAcc, Nucleus Accumbens; JOLO, Benton Judgement of Line Orientation – 15 item version; HVLT-R, Hopkins Verbal Learning Test- Revised; SF, Semantic Fluency; SDM, Symbol Digits Modalities Test; LNS, Letter Number Sequencing; Carriers, PD APOE4 carriers; Non-carriers, PD APOE4 non-carriers. a P-values are reported as uncorrected, with a p-value threshold of 0.05 (statistical significance in bold). (DOCX) [file pone.0341240.s002.docx]

**Supplementary Table 2: Correlation analyses between cognitive scores and gray matter volume of left angular gyrus and left nucleus accumbens in the whole PD cohort and subgroup analyses.**

| **Brain Region** | **Group** | **Cognitive Test** | **Pearson Correlation Coefficient (r)** | **p-value^a^** |
| --- | --- | --- | --- | --- |
| Lh ANG | Whole PD Cohort | **JOLO** | **0.267** | **< 0.001** |
|  |  | HVLT-Total Recall | -0.014 | 0.861 |
|  |  | HVLT- Delayed Recall | -0.037 | 0.633 |
|  |  | HVLT-Retention | -0.083 | 0.283 |
|  |  | HVLT-Discrimination | -0.064 | 0.407 |
|  |  | SF (Animals) | 0.026 | 0.736 |
|  |  | SDM | -0.024 | 0.754 |
|  |  | LNS | 0.126 | 0.102 |
|  | Carriers | **JOLO** | **0.323** | **0.021** |
|  | Non-carriers | **JOLO** | **0.239** | **0.009** |
| Lh NAcc | Whole PD Cohort | **JOLO** | **0.230** | **0.003** |
|  |  | HVLT-Total Recall | -0.099 | 0.200 |
|  |  | HVLT- Delayed Recall | -0.027 | 0.731 |
|  |  | HVLT-Retention | 0.015 | 0.844 |
|  |  | HVLT-Discrimination | -0.142 | 0.066 |
|  |  | SF (Animals) | -0.029 | 0.705 |
|  |  | SDM | 0.065 | 0.404 |
|  |  | **LNS** | **0.200** | **0.009** |
|  | Carriers | JOLO | 0.144 | 0.315 |
|  | Non-carriers | **JOLO** | **0.271** | **0.003** |
|  | Carriers | LNS | 0.171 | 0.231 |
|  | Non-carriers | **LNS** | **0.219** | **0.017** |

Analysis was first conducted in the whole cohort to identify significant correlations. Subgroup analyses (PD *APOE4* carriers and PD *APOE4* non-carriers) were then performed for cognitive tests that showed significant correlations in the whole group analyses. Significant correlations are bolded. Horizontal lines indicate the transition from whole cohort results to subgroup analyses. Abbrevations: Lh, left hemisphere; ANG, Angular Gyrus; NAcc, Nucleus Accumbens; JOLO, Benton Judgement of Line Orientation - 15 item version; HVLT-R Hopkins Verbal Learning Test- Revised; SF, Semantic Fluency; SDM, Symbol Digits Modalities Test; LNS, Letter Number Sequencing. Carriers, PD *APOE4* carriers; Non-carriers, PD *APOE4* non-carriers

^a^ P-values are reported as uncorrected, with a p-value threshold of 0.05 (statistical significance in bold).
