# Supplementary material for: The effect of Apolipoprotein E4 on cognitive function in Parkinson’s disease: A structural MRI study in the PPMI cohort
Source: PLoS One. 2026 Jan 20;21(1):e0341240. doi: 10.1371/journal.pone.0341240 (PMC12818682; doi:10.1371/journal.pone.0341240)
Supplement: S3 Table — Analysis of significant associations between left AnG GMV and JOLO scores when adjusting for age, sex, disease duration, and eTIV. Significant associations after co-variate adjustment are bolded. Abbreviations: Lh, left hemisphere; GMV, gray matter volume; eTIV, estimated total intracranial volume; AnG; Angular Gyrus; JOLO, Benton Judgement of Line Orientation – 15 item version; CI, Confidence Interval; β, Beta Coefficient; SE, Standard Error. a P-values are reported as uncorrected, with a p-value threshold of 0.05 (statistical significance in bold). (DOCX) [file pone.0341240.s003.docx]

**Supplementary Table 3: Regression models adjusted for covariates to examine the association between left angular gyrus gray matter volume and JOLO scores for whole PD cohort and subgroup analyses.**

| **Group** | **Variable** | **β** | **SE** | **CI (Lower)** | **CI (Upper)** | **p-value^a^** | **R**^2^ | **Adjusted R**^2^ |
| --- | --- | --- | --- | --- | --- | --- | --- | --- |
| Whole Group | Intercept | 3.696 | 2.946 | -2.12128 | 9.51405 | 0.211 | 0.230 | 0.207 |
|  | Lh AnG GMV | -0.000 | 0.000 | -0.00034 | 0.00025 | 0.764 | 0.230 | 0.207 |
|  | Age | **-0.054** | **0.023** | **-0.09981** | **-0.00916** | **0.019** | **0.230** | **0.207** |
|  | Sex(M) | 0.262 | 0.529 | -0.78293 | 1.30754 | 0.621 | 0.230 | 0.207 |
|  | Disease Duration | -0.006 | 0.014 | -0.03503 | 0.02210 | 0.655 | 0.230 | 0.207 |
|  | eTIV | **0.000** | **0.000** | **0.00000** | **0.00001** | **< 0.001** | **0.230** | **0.207** |
| *APOE4* Carriers | Intercept | 5.443 | 6.573 | -7.79606 | 18.68130 | 0.412 | 0.180 | 0.089 |
|  | Lh AnG GMV | 0.000 | 0.000 | -0.00031 | 0.00088 | 0.337 | 0.180 | 0.089 |
|  | Age | -0.052 | 0.054 | -0.16000 | 0.05688 | 0.343 | 0.180 | 0.089 |
|  | Sex(M) | 0.645 | 1.178 | -1.72700 | 3.01682 | 0.587 | 0.180 | 0.089 |
|  | Disease Duration | 0.045 | 0.045 | -0.04504 | 0.13554 | 0.318 | 0.180 | 0.089 |
|  | eTIV | 0.000 | 0.000 | -0.00000 | 0.00001 | 0.384 | 0.180 | 0.089 |
| *APOE4* Non-Carriers | Intercept | 3.118 | 3.205 | -3.23315 | 9.46886 | 0.333 | 0.312 | 0.282 |
|  | Lh AnG GMV | -0.000 | 0.000 | -0.00057 | 0.00012 | 0.196 | 0.312 | 0.282 |
|  | Age | -0.073 | 0.025 | -0.12214 | -0.02317 | 0.004 | 0.312 | 0.282 |
|  | Sex (M) | -0.232 | 0.593 | -1.40746 | 0.94333 | 0.696 | 0.312 | 0.282 |
|  | Disease Duration | -0.017 | 0.014 | -0.04596 | 0.01142 | 0.235 | 0.312 | 0.282 |
|  | eTIV | **0.000** | **0.000** | **0.00001** | **0.00002** | **< 0.001** | **0.312** | **0.282** |

Analysis of significant associations between left AnG GMV and JOLO scores when adjusting for age, sex, disease duration, and eTIV. Significant associations after co-variate adjustment are bolded. Abbreviations: Lh, left hemisphere; GMV, gray matter volume; eTIV, estimated total intracranial volume; AnG; Angular Gyrus; JOLO, Benton Judgement of Line Orientation - 15 item version; CI, Confidence Interval, β, Beta Coefficient; SE, Standard Error.

^a^ P-values are reported as uncorrected, with a p-value threshold of 0.05 (statistical significance in bold).
