# Supplementary material for: The effect of Apolipoprotein E4 on cognitive function in Parkinson’s disease: A structural MRI study in the PPMI cohort
Source: PLoS One. 2026 Jan 20;21(1):e0341240. doi: 10.1371/journal.pone.0341240 (PMC12818682; doi:10.1371/journal.pone.0341240)
Supplement: S4 Table — Data presented as adjusted mean (Standard Error) in mm3. All statistical tests were adjusted for age, sex, disease duration, and eTIV as co-variates. Abbrevations: Lh, left hemisphere; Rh, right hemisphere; eTIV, estimated total intracranial volume; ACC, Anterior Cingulate; Thal, Thalamus; Amyg, Amygdala; Caud, Caudate; NAcc, Nucleus Accumbens; EC, Entorhinal Cortex. a P-values are reported as uncorrected, with a p-value threshold of 0.05. No p-values are emphasized, as none survived Bonferroni correction for multiple comparison correction (p > 0.004). (DOCX) [file pone.0341240.s004.docx]

**Supplementary Table 4: Adjusted group comparisons of gray matter volume across exploratory regions between PD *APOE4* carriers and non-carriers.**

| **Region of Interest** | **Adjusted mean (non-carriers)** | **Adjusted mean**  **(carriers)** | **P**  **value**^a^ | **R^2^**  **adjusted** | **η2**  **partial** | **F**  **value** |
| --- | --- | --- | --- | --- | --- | --- |
| Lh ACC | 1629.422(37.752) | 1655.717(59.142) | 0.704 | 0.144 | 0.001 | 0.144 |
| Lh Thal | 7265.560(54.116) | 7271.676(84.777) | 0.951 | 0.626 | 0.000 | 0.004 |
| Lh Caud | 3383.283(36.962) | 3388.583(57.904) | 0.938 | 0.274 | 0.000 | 0.006 |
| Lh Amyg | 1693.984(20.054) | 1633.969(31.416) | 0.104 | 0.347 | 0.016 | 2.665 |
| Lh NAcc | 499.53(8.192) | 462.639(12.834) | 0.015 | 0.293 | 0.035 | 6.034 |
| Lh EC | 1900.967(34.911) | 1944.426(54.691) | 0.498 | 0.158 | 0.003 | 0.461 |
| Rh ACC | 1945.623(39.682) | 1952.305(62.165) | 0.927 | 0.184 | 0.000 | 0.008 |
| Rh Thal | 7310.981(52.604) | 7275.139(82.408) | 0.711 | 0.638 | 0.001 | 0.138 |
| Rh Caud | 3494.971(37.729) | 3513.315(59.106) | 0.791 | 0.280 | 0.000 | 0.070 |
| Rh Amyg | 1766.785(17.404) | 1756.506(27.265) | 0.748 | 0.496 | 0.001 | 0.104 |
| Rh NAcc | 527.861(8.609) | 513.913(13.487) | 0.378 | 0.260 | 0.005 | 0.781 |
| Rh EC | 1880.895(32.331) | 1848.272(50.650) | 0.583 | 0.140 | 0.002 | 0.303 |

Data presented as adjusted mean (Standard Error) in mm^3^. All statistical tests were adjusted for age, sex, disease duration, and eTIV as co-variates. Abbrevations: Lh, left hemisphere; Rh, right hemisphere; eTIV, estimated total intracranial volume; ACC, Anterior Cingulate; Thal, Thalamus; Amyg, Amygdala; Caud, Caudate; NAcc, Nucleus Accumbens; EC, Entorhinal Cortex.

^a^ P-values are reported as uncorrected, with a p-value threshold of 0.05. No p-values are emphasized, as none survived Bonferroni correction for multiple comparison correction (p > 0.004).
