# Supplementary material for: The effect of Apolipoprotein E4 on cognitive function in Parkinson’s disease: A structural MRI study in the PPMI cohort
Source: PLoS One. 2026 Jan 20;21(1):e0341240. doi: 10.1371/journal.pone.0341240 (PMC12818682; doi:10.1371/journal.pone.0341240)
Supplement: S5 Table — Analysis of significant associations between left NAcc GMV and JOLO scores when adjusting for age, sex, disease duration, and eTIV. Significant associations after co-variate adjustment are bolded. Abbreviations: Lh, left hemisphere; GMV, gray matter volume; eTIV, estimated total intracranial volume; NAcc, Nucleus Accumbens; JOLO, Benton Judgement of Line Orientation – 15 item version; CI, Confidence Interval; β, Beta Coefficient; SE, Standard Error. a P-values are reported as uncorrected, with a p-value threshold of 0.05 (statistical significance in bold). (DOCX) [file pone.0341240.s005.docx]

**Supplementary Table 5: Regression models adjusted for covariates to examine the association between the left nucleus accumbens gray matter volume and JOLO scores for whole PD cohort and subgroup analyses.**

| **Group** | **Variable** | **(β)** | **SE** | **CI (Lower)** | **CI (Upper)** | **p-value^a^** | **R**^2^ | **Adjusted R**^2^ |
| --- | --- | --- | --- | --- | --- | --- | --- | --- |
| Whole Group | Intercept | 2.442 | 3.220 | -3.91635 | 8.79965 | 0.449 | 0.233 | 0.209 |
|  | Lh NAcc GMV | 0.002 | 0.002 | -0.00268 | 0.00629 | 0.427 | 0.233 | 0.209 |
|  | Age | -0.043 | 0.025 | -0.09174 | 0.00591 | 0.084 | 0.233 | 0.209 |
|  | Sex(M) | 0.311 | 0.517 | -0.70842 | 1.33140 | 0.547 | 0.233 | 0.209 |
|  | Disease Duration | -0.006 | 0.014 | -0.03429 | 0.02288 | 0.694 | 0.233 | 0.209 |
|  | eTIV | **0.000** | **0.000** | **0.00000** | **0.00001** | **< 0.001** | **0.233** | **0.209** |
| *APOE4* Carriers | Intercept | 5.681 | 7.446 | -9.31544 | 20.67810 | 0.449 | 0.163 | 0.070 |
|  | Lh NAcc GMV | 0.001 | 0.006 | -0.01054 | 0.01292 | 0.839 | 0.163 | 0.070 |
|  | Age | -0.056 | 0.060 | -0.17666 | 0.06455 | 0.354 | 0.163 | 0.070 |
|  | Sex(M) | 0.597 | 1.192 | -1.80378 | 2.99714 | 0.619 | 0.163 | 0.070 |
|  | Disease Duration | 0.045 | 0.046 | -0.04658 | 0.13715 | 0.326 | 0.163 | 0.070 |
|  | eTIV | 0.000 | 0.000 | -0.00000 | 0.00001 | 0.138 | 0.163 | 0.070 |
| *APOE4* Non-Carriers | Intercept | 1.483 | 3.564 | -5.57811 | 8.54406 | 0.678 | 0.305 | 0.274 |
|  | Lh NAcc GMV | 0.002 | 0.002 | -0.00309 | 0.00648 | 0.484 | 0.305 | 0.274 |
|  | Age | -0.052 | 0.028 | -0.10701 | 0.00264 | 0.062 | 0.305 | 0.274 |
|  | Sex(M) | 0.008 | 0.566 | -1.11392 | 1.13068 | 0.988 | 0.305 | 0.274 |
|  | Disease Duration | -0.017 | 0.015 | -0.04609 | 0.01172 | 0.241 | 0.305 | 0.274 |
|  | eTIV | **0.000** | **0.000** | **0.00000** | **0.00001** | **< 0.001** | **0.305** | **0.274** |

Analysis of significant associations between left NAcc GMV and JOLO scores when adjusting for age, sex, disease duration, and eTIV. Significant associations after co-variate adjustment are bolded. Abbreviations: Lh, left hemisphere; GMV, gray matter volume; eTIV, estimated total intracranial volume; NAcc, Nucleus Accumbens; JOLO, Benton Judgement of Line Orientation - 15 item version; CI, Confidence Interval; β, Beta Coefficient; SE, Standard Error.

^a^ P-values are reported as uncorrected, with a p-value threshold of 0.05 (statistical significance in bold).
