# Supplementary material for: The effect of Apolipoprotein E4 on cognitive function in Parkinson’s disease: A structural MRI study in the PPMI cohort
Source: PLoS One. 2026 Jan 20;21(1):e0341240. doi: 10.1371/journal.pone.0341240 (PMC12818682; doi:10.1371/journal.pone.0341240)
Supplement: S6 Table — Analysis of significant associations between left NAcc GMV and LNS scores when adjusting for age, sex, disease duration, and eTIV. Significant associations after co-variate adjustment are bolded. Abbreviations: Lh, left hemisphere; eTIV, estimated total intracranial volume; NAcc, Nucleus Accumbens; LNS, Letter Number Sequencing Scores; CI, Confidence Interval; β, Beta Coefficient; SE, Standard Error. a P-values are reported as uncorrected, with a p-value threshold of 0.05 (statistical significance in bold). (DOCX) [file pone.0341240.s006.docx]

**Supplementary Table 6: Regression models adjusted for covariates to examine the association between the left nucleus accumbens gray matter volume and LNS scores for whole PD cohort and subgroup analyses.**

| **Group** | **Variable** | **(β)** | **SE** | **CI (Lower)** | **CI (Upper)** | **p-value^a^** | **R**^2^ | **Adjusted R**^2^ |
| --- | --- | --- | --- | --- | --- | --- | --- | --- |
| Whole Group | Intercept | 4.335 | 3.280 | -2.14197 | 10.81255 | 0.188 | 0.096 | 0.069 |
|  | Lh NAcc GMV | 0.004 | 0.002 | -0.00072 | 0.00842 | 0.098 | 0.096 | 0.069 |
|  | Age | -0.018 | 0.025 | -0.06786 | 0.03162 | 0.473 | 0.096 | 0.069 |
|  | Sex(M) | -0.975 | 0.526 | -2.01390 | 0.06419 | 0.066 | 0.096 | 0.069 |
|  | Disease Duration | 0.027 | 0.015 | -0.00238 | 0.05587 | 0.072 | 0.096 | 0.069 |
|  | eTIV | **0.000** | **0.000** | **0.00000** | **0.00001** | **0.009** | **0.096** | **0.069** |
| *APOE4* Carriers | Intercept | 4.458 | 5.902 | -7.42859 | 16.34485 | 0.454 | 0.141 | 0.046 |
|  | Lh NAcc GMV | 0.001 | 0.005 | -0.00801 | 0.01059 | 0.782 | 0.141 | 0.046 |
|  | Age | -0.029 | 0.047 | -0.12467 | 0.06652 | 0.543 | 0.141 | 0.046 |
|  | Sex(M) | -1.342 | 0.945 | -3.24467 | 0.56063 | 0.162 | 0.141 | 0.046 |
|  | Disease Duration | 0.031 | 0.036 | -0.04150 | 0.10413 | 0.391 | 0.141 | 0.046 |
|  | eTIV | **0.000** | **0.000** | **0.00000** | **0.00001** | **0.033** | **0.141** | **0.046** |
| *APOE4* Non-Carriers | Intercept | 2.522 | 4.076 | -5.55435 | 10.59910 | 0.537 | 0.102 | 0.062 |
|  | Lh NAcc GMV | **0.006** | **0.003** | **0.00049** | **0.01143** | **0.033** | **0.102** | **0.062** |
|  | Age | 0.008 | 0.032 | -0.05436 | 0.07107 | 0.792 | 0.102 | 0.062 |
|  | Sex(M) | -0.966 | 0.648 | -2.25007 | 0.31739 | 0.139 | 0.102 | 0.062 |
|  | Disease Duration | 0.031 | 0.017 | -0.00236 | 0.06377 | 0.068 | 0.102 | 0.062 |
|  | eTIV | **0.000** | **0.000** | **-0.00000** | **0.00001** | **0.088** | **0.102** | **0.062** |

Analysis of significant associations between left NAcc GMV and LNS scores when adjusting for age, sex, disease duration, and eTIV. Significant associations after co-variate adjustment are bolded. Abbreviations: Lh, left hemisphere; estimated total intracranial volume; NAcc, Nucleus Accumbens; LNS, Letter Number Sequencing Scores; CI, Confidence Interval; β, Beta Coefficient; SE, Standard Error; eTIV,

^a^ P-values are reported as uncorrected, with a p-value threshold of 0.05 (statistical significance in bold).
