# Supplementary material for: The effect of Apolipoprotein E4 on cognitive function in Parkinson’s disease: A structural MRI study in the PPMI cohort
Source: PLoS One. 2026 Jan 20;21(1):e0341240. doi: 10.1371/journal.pone.0341240 (PMC12818682; doi:10.1371/journal.pone.0341240)
Supplement: S7 Table — Data presented as adjusted mean (Standard Error) in mm. All statistical tests were adjusted for age, sex, and disease duration as co-variates. Bold Horizontal line separates specific regions from exploratory regions. Abbreviations: Lh, left hemisphere; Rh, right hemisphere; noncarriers, PD APOE4 non-carriers; carriers, PD APOE4 carriers; ANG, Angular Gyrus; DLPFC, Dorsolateral Prefrontal Cortex; HPC, Hippocampus; INS, Insula; SFG, Superior Frontal Gyrus; STG, Superior Temporal Gyrus; SMG, Supramarginal Gyrus; ACC, Anterior Cingulate; EC, Entorhinal Cortex. a P-values are reported as uncorrected, with a p-value threshold of 0.05 (statistical significance in bold). (DOCX) [file pone.0341240.s007.docx]

**Supplementary Table 7: Adjusted group comparisons of cortical thickness across specific and exploratory regions between PD *APOE4* carriers and non-carriers.**

| **Region of Interest** | **Adjusted mean (non-carriers)** | **Adjusted mean**  **(carriers)** | **p-**  **value**^a^ | **R^2^**  **adjusted** | **η2**  **partial** | **F**  **value** |
| --- | --- | --- | --- | --- | --- | --- |
| Lh ANG | 2.338(0.009) | 2.334(0.014) | 0.826 | 0.148 | 0.000 | 0.048 |
| Lh DLPFC | 2.286(0.009) | 2.310(0.013) | 0.139 | -0.007 | 0.013 | 2.213 |
| Lh SFG | 2.540(0.009) | 2.561(0.014) | 0.228 | 0.084 | 0.009 | 1.464 |
| Lh STG | 2.633(0.012) | 2.618(0.018) | 0.498 | 0.187 | 0.003 | 0.461 |
| Lh SMG | 2.418(0.009) | 2.409(0.014) | 0.577 | 0.196 | 0.002 | 0.312 |
| Lh INS | 2.845(0.012) | 2.827(0.019) | 0.4 | 0.018 | 0.004 | 0.712 |
| Rh ANG | 2.384(0.008) | 2.381(0.0013) | 0.795 | 0.195 | 0.000 | 0.068 |
| Rh DLPFC | 2.269(0.009) | 2.280(0.014) | 0.512 | 0.010 | 0.003 | 0.432 |
| Rh SFG | 2.525 (0.009) | 2.542(0.015) | 0.332 | 0.000 | 0.016 | 0.946 |
| Rh STG | 2.657 (0.011) | 2.651(0.017) | 0.746 | 0.226 | 0.001 | 0.105 |
| Rh SMG | 2.440 (0.009) | 2.438(0.014) | 0.887 | 0.200 | 0.000 | 0.022 |
| Rh INS | 2.871 (0.013) | 2.870(0.02-) | 0.973 | 0.038 | 0.000 | 0.001 |
| Lh ACC | 2.346 (0.017) | 2.365(0.027) | 0.553 | 0.072 | 0.002 | 0.354 |
| Lh EC | 3.110 (0.024) | 3.099(0.037) | 0.797 | 0.093 | 0.000 | 0.066 |
| Rh ACC | 2.217 (0.015) | 2.233(0.024) | 0.562 | 0.013 | 0.002 | 0.338 |
| Rh EC | 3.218(0.028) | 3.178 (0.044) | 0.445 | 0.030 | 0.004 | 0.586 |

Data presented as adjusted mean (Standard Error) in mm. All statistical tests were adjusted for age, sex, and disease duration as co-variates. Bold Horizontal line separates specific regions from exploratory regions.

Abbreviations: Lh, left hemisphere; Rh, right hemisphere; noncarriers, PD *APOE4* non-carriers; carriers, PD *APOE4* carriers; ANG, Angular Gyrus; DLPFC, Dorsolateral Prefrontal Cortex; HPC, INS, Insula; SFG, Superior Frontal Gyrus; STG, Superior Temporal Gyrus; SMG, Supramarginal Gyrus; ACC, Anterior Cingulate; EC, Entorhinal Cortex.

^a^ P-values are reported as uncorrected, with a p-value threshold of 0.05 (statistical significance in bold).
