# Supplementary material for: The effect of Apolipoprotein E4 on cognitive function in Parkinson’s disease: A structural MRI study in the PPMI cohort
Source: PLoS One. 2026 Jan 20;21(1):e0341240. doi: 10.1371/journal.pone.0341240 (PMC12818682; doi:10.1371/journal.pone.0341240)
Supplement: S8 Table — Data presented as adjusted mean (Standard Error) in mm3. All statistical tests were adjusted for age, sex, disease duration, and eTIV as co-variates. Abbreviations: Lh, left hemisphere; Rh, right hemisphere; eTIV, estimated total intracranial volume; Hipp, Hippocampal; noncarriers; PD APOE4 non-carriers; carriers, PD APOE4 carriers; CA, Cornu Ammonis; Molecular Layer, Molecular layer of the dentate gyrus; GC-ML-DG, Granule cell and molecular layer of the dentate gyrus. a P-values are reported as uncorrected, with a p-value threshold of 0.05 (statistical significance in bold). (DOCX) [file pone.0341240.s008.docx]

**Supplementary Table 8: Adjusted group differences of gray matter volume across hippocampal subregions between PD *APOE4* carriers and non-carriers.**

| **Hippocampal Subregion** | **Adjusted mean**  **(non-carriers)** | **Adjusted mean**  **(carriers)** | **p-**  **value**^a^ | **R^2^**  **adjusted** | **η2**  **partial** | **F**  **value** |
| --- | --- | --- | --- | --- | --- | --- |
| Lh Hipp Tail | 575.990 (7.163) | 576.653(11.221) | 0.960 | 0.208 | 0.000 | 0.003 |
| Lh Subiculum | 453.232(4.682) | 442.551(7.335) | 0.215 | 0.321 | 0.009 | 1.548 |
| Lh CA1 | 652.141(7.251) | 643.057(11.359) | 0.495 | 0.325 | 0.003 | 0.467 |
| Lh Hipp Fissure | 162.732(2.338) | 169.035(3.663) | 0.143 | 0.089 | 0.013 | 2.161 |
| Lh Presubiculum | 332.179(3.610) | 333.599(5.655) | 0.830 | 0.292 | 0.000 | 0.046 |
| Lh Parasubiculum | 74.795(1.373) | 73.738(2.150) | 0.675 | 0.164 | 0.001 | 0.176 |
| Lh Molecular Layer | 566.663(5.205) | 559.414(8.153) | 0.448 | 0.412 | 0.003 | 0.577 |
| Lh GC-ML-DG | 287.929(2.814) | 286.809(4.408) | 0.828 | 0.371 | 0.000 | 0.047 |
| Lh CA3 | 208.799(2.599) | 211.667(4.072) | 0.548 | 0.265 | 0.002 | 0.362 |
| Lh CA4 | 247.822(2.428) | 247.269(3.803) | 0.901 | 0.336 | 0.000 | 0.015 |
| Lh Fimbria | 78.385(1.688) | 76.013(2.644) | 0.444 | 0.229 | 0.004 | 0.588 |
| Lh HATA | 62.236 (0.851) | 62.014(1.333) | 0.887 | 0.241 | 0.000 | 0.020 |
| Rh Hipp Tail | 607.866(7.058) | 611.032(11.057) | 0.807 | 0.253 | 0.000 | 0.060 |
| Rh Subiculum | 445.264(4.313) | 444.448(6.756) | 0.918 | 0.342 | 0.000 | 0.011 |
| Rh CA1 | 685.487(6.768) | 682.501(10.603) | 0.810 | 0.412 | 0.000 | 0.058 |
| Rh Hipp Fissure | 165.021(2.102) | 169.760(3.293) | 0.221 | 0.137 | 0.009 | 1.512 |
| Rh Presubiculum | 309.866(3.516) | 315.474(5.508) | 0.385 | 0.299 | 0.005 | 0.759 |
| Rh Parasubiculum | 69.682(1.340) | 69.163(2.099) | 0.833 | 0.095 | 0.000 | 0.045 |
| Rh Molecular Layer | 580.886(5.048) | 578.649(7.907) | 0.809 | 0.447 | 0.000 | 0.058 |
| Rh GC-ML-DG | 296.861(2.845) | 297.478(4.457) | 0.906 | 0.382 | 0.000 | 0.014 |
| Rh CA3 | 229.463(2.735) | 232.474(4.285) | 0.549 | 0.284 | 0.002 | 0.361 |
| Rh CA4 | 255.558(2.518) | 256.887(3.944) | 0.774 | 0.328 | 0.001 | 0.083 |
| Rh Fimbria | 72.019(1.735) | 70.228(2.718) | 0.574 | 0.230 | 0.002 | 0.317 |
| Rh HATA | 63.811(0.874) | 64.080(1.369) | 0.867 | 0.336 | 0.000 | 0.028 |

Data presented as adjusted mean (Standard Error) in mm^3^. All statistical tests were adjusted for age, sex, disease duration, and eTIV as co-variates. Abbreviations: Lh, left hemisphere; Rh, right hemisphere; eTIV, estimated total intracranial volume; Hipp, Hippocampal; noncarriers; PD *APOE4* non-carriers; carriers, PD *APOE4* carriers; CA, Cornu Ammonis; Molecular Layer, Molecular layer of the dentate gyrus; GC-ML-DG, Granule cell and molecular layer of the dentate gyrus.

^a^ P-values are reported as uncorrected, with a p-value threshold of 0.05 (statistical significance in bold).
